# Identification to species level of live single microalgal cells from plankton samples with matrix-free laser/desorption ionization mass spectrometry

**DOI:** 10.1007/s11306-020-1646-7

**Published:** 2020-02-24

**Authors:** Tim U. H. Baumeister, Marine Vallet, Filip Kaftan, Laure Guillou, Aleš Svatoš, Georg Pohnert

**Affiliations:** 1 Max Planck Institute for Chemical Ecology, Max Planck Fellow Group On Plankton Community Interaction, Hans-Knöll-Str. 8, 07745 Jena, Germany; 2 Research Group Mass Spectrometry/Proteomics, Max Planck Institute for Chemical Ecology, Hans-Knöll-Str. 8, 07745 Jena, Germany; 3 Sorbonne Université, CNRS, UMR7144 Adaptation Et Diversité en Milieu Marin, Ecology of Marine Plankton (ECOMAP), Station Biologique de Roscoff SBR, 29680 Roscoff, France; 4 Department of Bioorganic Analytics, Institute for Inorganic and Analytical Chemistry, Friedrich Schiller University Jena, Lessingstr. 8, 07743 Jena, Germany

**Keywords:** Microalgal identification, Live single-cell mass spectrometry, Matrix-free laser desorption/ionization high-resolution mass spectrometry, Spectral pattern matching, Spectrum similarity, Metabolic fingerprinting

## Abstract

**Introduction:**

Marine planktonic communities are complex microbial consortia often dominated by microscopic algae. The taxonomic identification of individual phytoplankton cells usually relies on their morphology and demands expert knowledge. Recently, a live single-cell mass spectrometry (LSC-MS) pipeline was developed to generate metabolic profiles of microalgae.

**Objective:**

Taxonomic identification of diverse microalgal single cells from collection strains and plankton samples based on the metabolic fingerprints analyzed with matrix-free laser desorption/ionization high-resolution mass spectrometry.

**Methods:**

Matrix-free atmospheric pressure laser-desorption ionization mass spectrometry was performed to acquire single-cell mass spectra from collection strains and prior identified environmental isolates. The computational identification of microalgal species was performed by spectral pattern matching (SPM). Three similarity scores and a bootstrap-derived confidence score were evaluated in terms of their classification performance. The effects of high and low-mass resolutions on the classification success were evaluated.

**Results:**

Several hundred single-cell mass spectra from nine genera and nine species of marine microalgae were obtained. SPM enabled the identification of single cells at the genus and species level with high accuracies. The receiver operating characteristic (ROC) curves indicated a good performance of the similarity measures but were outperformed by the bootstrap-derived confidence scores.

**Conclusion:**

This is the first study to solve taxonomic identification of microalgae based on the metabolic fingerprints of the individual cell using an SPM approach.

**Electronic supplementary material:**

The online version of this article (10.1007/s11306-020-1646-7) contains supplementary material, which is available to authorized users.

## Introduction

Phytoplankton inhabiting aquatic ecosystems worldwide is highly complex and can contain thousands of interacting species, which coexist and potentially compete for the same resources (Poulin et al. 2019; Schwartz et al. 2016). The classical taxonomic identification of larger phototrophic microalgae over 20 µm is based on morphological features of the cells that are investigated by light or electron microscopy (Hoppenrath et al. 2009). The identification requires expert knowledge and often leads to misclassifications due to morphological similarities of different species. Automated approaches, such as PlanktoVision have been developed and use a neural network that analyzes light-microscope pictures to classify microalgae (Schulze et al. 2013). More advanced workflows combine the algorithm-assisted picture analysis with fluorescence information, and yield taxonomic resolution on a single-cell level, even deriving the growth phase of the analyzed microalgae (Dunker et al. 2018). This approach has been applied for identification based on features from morphological criteria (e.g. ornamentation, contour, shape) or image characteristics (e.g. transparency, area) of living algal cells (Sosik and Olson 2007; Zheng et al. 2017) or purified diatom cell walls (Bueno et al. 2017). Complementary molecular genetic methods determining the taxonomic identity of algae are available since the 1970s and include now the genome sequencing of single microbial cells (Stepanauskas 2012). Nevertheless, most of these existing methods require time-consuming sample preparation and do not give information on the physiological status of cells, nor on cellular metabolites.

Matrix-assisted laser desorption-ionization time-of-flight mass spectrometry (MALDI-TOF MS) allows the identification of microorganisms such as bacteria, fungi, and algae, based on mass spectra of protein extracts from groups of cells (Barbano et al. 2015; Crossay et al. 2017; Mello et al. 2017; Murugaiyan and Roesler 2017). MALDI-TOF mass spectra analysis is established for the automated identification of bacteria by spectral pattern matching (SPM) and clustering algorithms, even when consortia of organisms were analyzed (Sandrin and Demirev 2018; Yang et al. 2018).

However, a group of cells, even sampled from a single clonal culture, is far from being homogeneous. Cells of the same population with very different physiological status can coexist (e.g. mitotically dividing, encysting, sexually reproducing, actively growing, resisting against pathogen). Working at the single cell level is the only way to explore both the identity and the physiology of a specimen. Single-cell analysis has been introduced in the last decade to profile the cellular content, providing information on the genes, proteome, transcriptome or metabolome of the organism under study (Yuan et al. 2017). Single-cell mass spectrometry of algal cells has already been developed with matrix-free laser desorption-ionization using the ionization-enhancing effect of diatom cell walls (Jaschinski et al. 2014). Live single-cell mass spectrometry (LSC-MS) with laser-desorption ionization high-resolution mass spectrometry was developed to profile reliably the low-molecular-weight metabolites of living cells kept in their native environment prior to analysis. This allows the study of the cellular physiology in microalgae (Baumeister et al. 2019). High-resolution atmospheric pressure scanning microprobe laser desorption/ionization mass spectrometry with its high spatial resolution (10 µm) enables individual targeting of intact live microbial cells under ambient conditions. Coupling of the source to an Orbitrap mass spectrometer provides high-resolution mass spectra (Baumeister et al. 2019; Schober et al. 2012). The analysis of such high-throughput data is however challenging. To date, only a few bioinformatic classification tools are available for (MA)LDI derived workflows, including the Bruker Biotyper® (Bizzini and Greub 2010), VITEK® MS (Branda et al. 2014) and the freeware Matlab based tool MicrobeMS (Lasch 2015). Recently, Yang et al. 2017 introduced an optimized SPM pipeline, whereby the reliability of the prediction was boosted by confidence scores that were derived from the identification results of bootstrap spectra from the query spectrum (Yang et al. 2017). This study aimed to demonstrate the utility of LSC-MS for the taxonomic discrimination of live single cells picked from natural samples. Therefore, we combined LSC-MS to generate single-cell mass spectra with the optimized SPM approach to enable microalgal cell identification, based solely on single-cell mass spectrometry profiles.

## Materials and methods

### Sampling and identification of microalgae

**Fig. 1:**
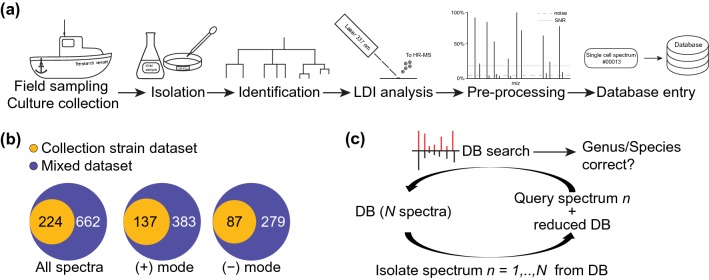
Workflow of the spectral database generation and data analysis using SPM. **a** Microalgae were obtained from culture collection and field sampling during the bloom season. Microalgal cells from the field samplings were isolated and identified. Single algal cells were analyzed in their native environment with matrix-free laser desorption/ionization high-resolution mass spectrometry. After pre-processing, each single-cell spectrum received a unique identification number and was recorded in the single-cell profile database. **b** Structure and content of the datasets analyzed with SPM: the collection strain dataset is a subset of the mixed dataset which includes single-cell spectra from field sampling algae. **c** Principle of the SPM, from the database (DB) containing *N* spectra, each spectrum (*n*) was once isolated and used as a query against the reduced DB

Algae strains were purchased from the Roscoff Culture Collection (RCC, Vaulot et al. 2004) and maintained under fluorescent lamps (irradiance 100 mE m^−2^ s^−1^) with a 14 h photoperiod coupled to a thermo-regulated cycle (16–12 °C day–night). Novel microalgal isolates were obtained during field sampling at the bloom season in the waters of Penzé Estuary (France, June 2018), Lesvos (Greece, May 2018), Helgoland (Germany, August 2016 and 2017) and Farsund (Norway, September 2017). The workflow from algal cell isolation to single-cell analysis is detailed in Fig. 1a. The field samples were concentrated by filtration using nylon mesh (40, 70 and 100 µm pore size, Corning Life Sciences) and washed with sterile-filtered seawater. Single algal cells were picked by micromanipulation under a binocular stereomicroscope (binocular VisiScope®, VWR International GmbH, Pennsylvania, US). Cells were transferred into Petri dishes with 5 mL sterile natural seawater (ATI, Gebesee, Germany). Single cells were then either re-isolated by pipetting and/or purified by dilution (Andersen 2005). Almost all of the isolated cells divided and multiplied to give sufficient individuals for analysis within 15 days. Meanwhile, cultures were visualized with light microscopy, photographed, and their morphological characteristics were compared with descriptions from previous studies of phytoplankton blooms in these areas (Hoppenrath et al. 2009). The isolated algal cells were identified to the genus level using light microscopy. All algae from field samplings and culture collection were maintained under the same culture conditions and grown in Guillard’s (F/2) enrichment medium (Sigma-Aldrich, Munich, Germany) prepared with natural 0.2 µm filtered and autoclaved seawater. Among the algal isolates, 15 were deposited in the RCC collection under strain numbers RCC6807 to RCC6821. Pictures of cells were taken with a 20 × /0.4 Ph2-Korr-Achroplan on an Axiovert 200 microscope (Zeiss, Jena, Germany).

### LSC-MS analysis and pre-processing of single-cell spectra

Single algal cells growing in the replete medium at the early growth stage were manually collected with a 20 µL pipette and deposited onto a GF/C glass fiber filter wetted with medium (Whatman, Maidstone, United Kingdom) according to Baumeister et al. (2019). For chain-forming diatoms such as *Chaetoceros* spp. or *Thalassiosira* spp., only cells separated from chains were analyzed, since the spatial resolution of the laser does not allow an analysis of single cells in chains. The AP-SMALDI ion source (TransMIT, Gießen, Germany) was coupled to a Q Exactive™ Plus (Thermo Fisher Scientific, Bremen, Germany) mass spectrometer to record high-resolution LSC-MS spectra. Individual cells were either analyzed in positive or negative polarity with 120 cycles per cell (1 min acquisition time). One cycle comprises 30 laser shots with a frequency of 60 Hz with an approximate energy of 1.5 μJ per shot. Mass spectra were recorded in the mass range from *m*/*z* 100 to 1000 with a resolving power of 280 000 (at *m*/*z *200). This range was chosen to cover a sufficient amount of metabolites for fingerprinting but it could be extended if metabolites of interest fall outside of the range. Every single-cell mass spectrum represents an average of all scans from the one-minute data acquisition. MS raw files were converted with the Thermo File Converter from the Xcalibur suite 3.0.63 to the netCDF format and processed with the MALDIquant R package (Gibb and Strimmer 2012). Sample spectra were de-noised (signal-to-noise ratio 5) and peaks co-occurring in medium blanks acquired from the sterile medium were removed. Processed spectra were conserved in the comma-separated values (CSV) file format. Upfront similarity scoring, spectra were normalized to the most abundant signal in the individual spectrum (base peak normalization). Integer mass spectra were generated from the high-resolution mass spectra by rounding the *m*/*z* values to integers and summing up the corresponding intensities of those signals that matched together after rounding. Datasheets with metadata were created (data files S11–S14), and a unique identifier (ID) was assigned to each LSC-MS profile. The metadata files contain information about the sampling site, date of isolation, date of LSC-MS analysis, growth medium used, and strain availability in a culture collection. The dataset structure and content are presented in Fig. 1b. Spectra, R scripts, metadata files and result data files used in this study are available in the Pohnert-Lab GitHub repository (https://github.com/Pohnert-Lab/SC-MS-Identification).

### Spectral similarity matching and statistical analysis

Data analysis was conducted in R 3.4.2 (R Core Team 2017). Normalization, similarity scoring, and bootstrap assessment (n = 500) of top hits from spectral pattern matching (SPM) were performed based on a method established by Yang et al. (2017). Mass tolerance for matching of high-resolution masses was set to ± 5 ppm, and for integer masses to ± 500 ppm. SPM of live single-cell mass spectra was performed with three similarity measures, the cosine correlation (*Cos*), the relative Euclidean distance similarity (*Eu*) and the intensity-weighted relative Euclidean distance similarity (*iEu*). Each spectrum of the in-house database was removed once from the database and used as the query (Fig. 1b). Only the match result with the highest score (top hit) was used for data evaluation. Sensitivities and error rates were calculated according to Yang et al. (2017), corresponding threshold scores were determined and the receiver operating characteristic (ROC) curves produced using the pROC package 1.10.0 (Robin et al. 2011). Confusion matrices were produced using the ModelMetrics package 1.1.0 (Hunt 2016). A qualitative rating of the AUC values was established according to Xia et al. (2013). The plots displaying the number of peaks per genus or species (Figs. S2, S3) and the plots showing the frequencies of peaks per spectrum (bin size *m*/*z* 10) per genus or species (Figs. S4–S7) were produced with the ggplot2 package 2.2.1 (Hadley 2016). Graphics were processed in Adobe Illustrator CS5.

## Results and discussion

### Microalgae samplings and LSC-MS acquisition

Microalgae belonging to the group of bloom-forming single-cell eukaryotes, coexisting in marine ecosystems, were selected for the study. Several diatom genera and one dinoflagellate genus were obtained from monoclonal public collection strains and from field samplings at many different locations in Europe. The established workflow, from algae isolation, maintenance in culture to single-cell analysis is depicted in Fig. 1a. Single algal cells from the field samplings were purified by dilution, photographed under a light microscope (Fig. S1), and identified based on morphological characteristics and literature (Hoppenrath et al. 2009). A high number of strains, mainly belonging to the genera *Guinardia*, *Coscinodiscus,* and *Chaetoceros* were recovered (Table S1). The process of obtaining one mass spectrum of one cell, including sample preparation, data acquisition to computation can be performed within few minutes. An important advantage of the method introduced here, compared to competing techniques is the low effort in sample preparation, which involves only filtration. Also the possibility to rely on data bases for identification once expert knowledge has been put in to classify the species is superior compared to traditional light microscopy approaches. High-resolution single-cell mass spectra of intact live algal cells were acquired in negative and positive polarity (Table S1). The overall data set, denoted as the "mixed dataset", contained 662 mass spectra acquired in positive (383 spectra) or negative polarity (279 spectra), obtained from 64 strains of 9 genera (Fig. 1b, Table S1). A subset of the whole dataset, referred to as the "collection strain dataset", consisted of spectra obtained from 9 species from culture collections. The collection strain dataset contained 224 spectra, including 137 single-cell spectra acquired in positive polarity and 87 spectra in negative polarity (Fig. 1b). To first assess the SPM methodology a dataset that contains only spectra from cells unambiguously identified to the species level was used. Later the mixed species datasets with more genera or isolates from the field were analyzed. Most of the spectra were rich in peaks but the number of peaks per cell was dependent on the individual and varied according to genus and species (Figs. S2, S3). The total count varied in the range from less than ten to several thousand peaks per spectrum. The absolute count of peaks tended to be higher in spectra obtained in positive polarity, with *Thalassiosira* being an exception (Fig. S2). The spectra were not further filtered, nor were peaks removed, as it is the practice in the generation of MALDI reference spectra of bacteria (Freiwald and Sauer 2009).

Frequencies of *m*/*z* values per spectrum (bin size *m*/*z* 10, split by genus and species) showed a similar trimodal-type pattern (Figs. S4–S7), whereby the region in between *m*/*z* 100–330 usually contained most of the signals, followed by the range *m*/*z* 430–660 and few but pronounced signals were observed in the range of *m*/*z* 760–880. The following taxonomic identification approach relied entirely on the whole mass spectral fingerprint and therefore the underlying pattern produced by all detected signals. Nevertheless, the molecular classes that are principally addressed with this technique are noteworthy. It was shown that direct LDI-MS of intact microalgal cells addresses photosensitive molecules such as pigments (e.g. carotenes and chlorophyll), as well as lipids and even zwitterionic molecules such as DMSP (Urban et al. 2011; Baumeister et al. 2019).

### Identification of microalgae based on spectral pattern matching

**Fig. 2:**
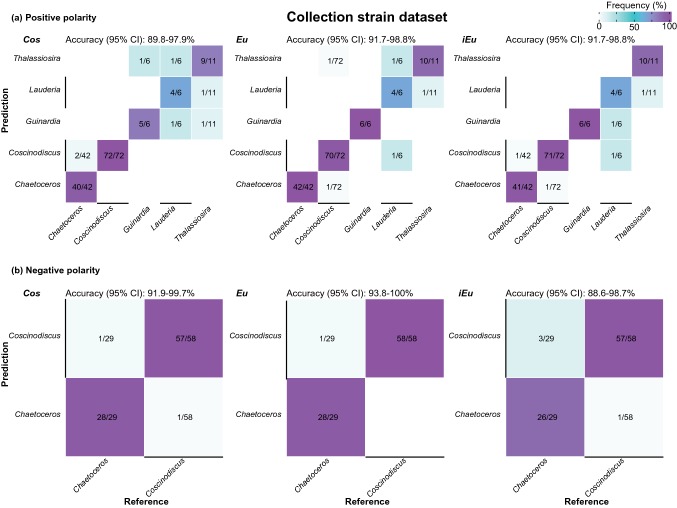
Confusion matrices of identification results of microalgae at the genus level by *Cos*, *Eu*, and *iEu*, for the collection strain dataset. **a** Underlying high-resolution single-cell spectra acquired in positive polarity. **b** Underlying high-resolution single-cell spectra acquired in negative polarity. Confidence intervals (95%) of overall accuracies are indicated above each plot

**Fig. 3:**
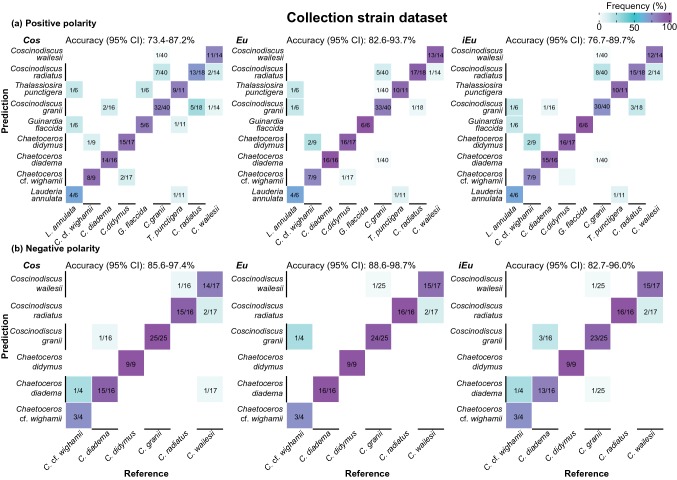
Confusion matrices of identification results of microalgae at the species level by *Cos*, *Eu*, and *iEu*, for the collection strain dataset. **a** Underlying high-resolution single-cell spectra acquired in positive polarity. **b** Underlying high-resolution single-cell spectra acquired in negative polarity. Confidence intervals (95%) of overall accuracies are indicated above each plot

Each single-cell mass spectrum was used as a query for the SPM (Fig. 1c) and was therefore isolated from the database following the method from Yang et al. 2017. Identification success was evaluated independently for the respective polarity and spectral similarity measure. Results were visualized in confusion matrices (Figs. 2, 3, S8–S10). A confusion matrix gives an overview of the classification success (hits) and misclassification of the queried spectra dependent on the used classifier (Dunker et al. 2018).

The analysis of the collection strain dataset revealed convincing identification results, with overall accuracies in the range of 88.6% to 100% at the genus (Fig. 2) and 73.4% to 98.7% at the species level (Fig. 3). The similarity measures *Eu* and *iEu* performed slightly better than *Cos*, especially at the species level (Fig. 3), as did spectra obtained in negative polarity (Figs. 2, 3). However, the direct comparison of both polarities is challenging, since each cell could only be analyzed by one polarity. Misclassifications at the species level often occurred in the way that species were misclassified as a species from the same genus, as indicated by the very high accuracies of up to 100% at the genus level.

The mixed dataset which extended the collection strain dataset by cells from field sampling showed lower overall classification accuracies of 79.0% to 89.9% (Fig. S8). Especially *Pleurosigma* and *Rhizosolenia* were wrongly assigned quite often to various different genera. The differences in accuracy between the similarity measures and also the ionization polarities were negligible and small. Nevertheless, the obtained accuracies are in the same range as of taxonomical experts (Culverhouse et al. 2003) or machine learning approaches (Zheng et al. 2017) which assign the algal identity based on microscopy images.

### Statistical assessment of the SPM-driven identification

**Fig. 4:**
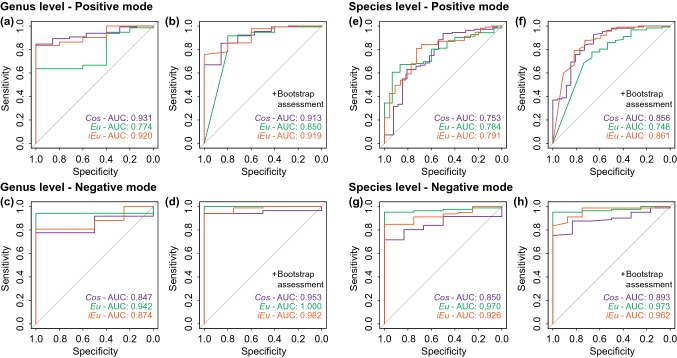
Receiver operating characteristic curves and corresponding areas under the curves (AUC) for the identification of single microalgal cells for the collection strain dataset. Assessment of identification at either the genus (**a**–**d**) or species levels (**e**–**h**). The analysis was performed with bootstrap assessment (**b**, **d**, **f**, **h**) or without (**a**, **c**, **e**, **g**). High-resolution single-cell spectra recovered from the positive (**a**, **b**, **e**, **f**) or negative (**c**, **d**, **g**, **h**) polarity were independently analyzed. The AUC curves obtained for each classifier (*Cos*, *Eu*, *iEu*) analyzed are indicated in purple, green or orange color, respectively

**Fig. 5:**
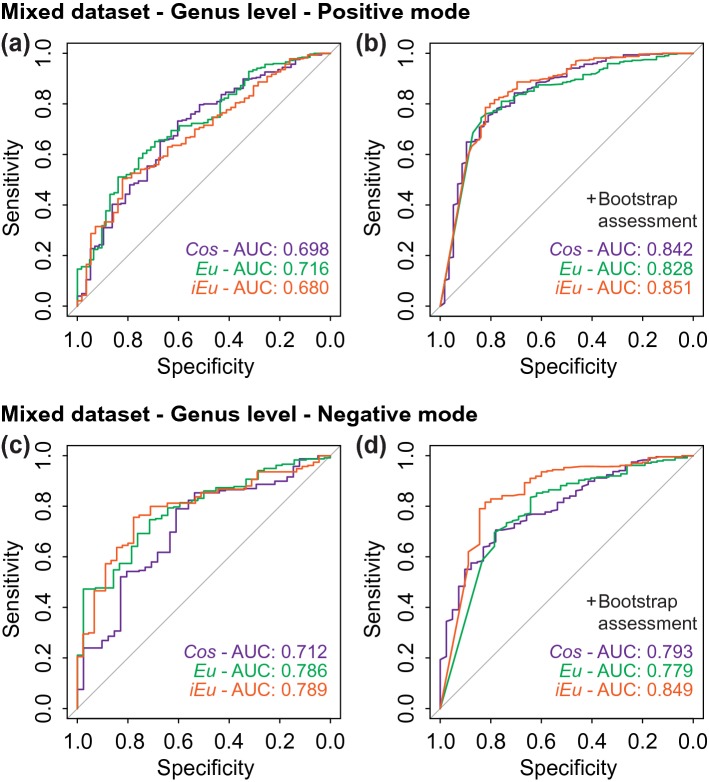
Receiver operating curves and corresponding areas under the curves (AUC) for the identification of single microalgal cells at the genus level for the mixed dataset comprising collection strains and field isolates. The analysis was performed with bootstrap assessment (**b**, **d**) or without (**a**, **c**). High-resolution single-cell spectra recovered from the positive (**a**, **b**) or negative (**c**, **d**) polarity were independently analyzed. The AUC curves obtained for each classifier (*Cos*, *Eu*, *iEu*) analyzed are indicated in purple, green or orange color, respectively

To evaluate the performance of the microalgal identification based on SPM, the receiver operating characteristic (ROC) curves were obtained for the three similarity measures and the bootstrap-derived confidence scores, further divided by dataset, genus or species level and polarity (Figs. 4, 5, S11, S12). The ROC curves illustrate the relationship between sensitivity (true positive rate) and specificity (true negative rate) of one or more classifiers by constantly altering the decision threshold (Zweig and Campbell 1993). In this study, the classifiers are the three similarity measures (*Cos*, *Eu, iEu*) and the corresponding bootstrap-derived confidence scores. The area under the ROC curve (AUC) is an established measure of performance of analyzed classifiers, whereby an area greater than that under the diagonal (AUC > 0.5) indicates a positive non-stochastical classification (Hanley and McNeil 1982).

We first evaluated the classifier performance at the genus and species level based on the collection strain dataset (Fig. 4). At the genus level, the best scores were obtained with *Cos* (AUC: 0.931) and *Eu* (AUC: 0.942) for single-cell spectra acquired in positive and negative polarity, respectively (Fig. 4a, c). Fair AUCs of 0.753 to 0.791 (positive polarity) and good to excellent AUCs of 0.850 to 0.970 (negative polarity) were reached at the species level (Fig. 4e, g). The scoring measures *Eu* and *iEu* surpassed *Cos* in all analyses, except for the spectra at the genus level in positive polarity (Fig. 4a). The classification performance dropped when the mixed dataset was used and poor to fair AUCs in the range of 0.680 to 0.789 were obtained (Fig. 5a, c). The bootstrap-dependent confidence scores improved the classification performance for most of the analyses, but especially for those that exhibited poor to fair AUCs when the similarity scores were used as the classifier (Fig. 4b, d, f, h, 5b, d).

Furthermore, we determined threshold scores and the corresponding sensitivities at error rates below a fixed value (Table S2). For example, error rates of less than 5%, sensitivities of up to 100% were achieved at the genus level using the collection strain dataset. The confidence scores yielded in general higher sensitivities than the similarity measures *Cos*, *Eu*, and *iEu* at the same error rate (Table S2). This finding is in accordance with the initial study by Yang et al. classifying bacterial species by protein mass spectra (Yang et al. 2017). Based on these results, it is recommended using the bootstrapping assessment for the classification of microalgae by single-cell mass spectrometry profiling and SPM.

### Assessment of the mass resolution on microalgal species identification

In proteomics, metabolomics, and related fields, high resolution and accuracy of the analyzed masses is a desired feature (Comi et al. 2017). Many database-driven SPM methods still rely on unit mass resolution spectra (Kwiecien et al. 2015; Stein 1999). SPM of bacterial spectra obtained with MALDI-TOF works with very high relative mass deviations (over 200 ppm) (Sauer and Kliem 2010). To evaluate if the single-cell identification depends solely on high mass resolution data, the spectra were rounded to unit mass resolution, referred then as integer mass spectra, and analyzed with the SPM workflow.

The number of peaks per genus and per species dropped substantially in some cases (Figs. S2, S3) and four spectra had to be removed from the database for *Chaetoceros* since only one peak remained in the respective spectra. However, the confusion matrices and ROC curves showed high similarities with those obtained from high-resolution spectra (Figs. S9–S12). Consequently, future acquisitions of mass spectra from microalgae could be performed at a lower resolution, which would allow the use of mass spectrometers with less resolving power, or, for Orbitrap measurements, to acquire more scans per time interval (Zubarev and Makarov 2013), hence increasing the sensitivity. Furthermore, SPM with integer mass spectra allows faster computation, since peak matching between reference and query spectrum is simplified as the mass deviation no longer has to be taken into account. However, it can be assumed that increasing the database with more algal genera and species will require more resolution to distinguish between taxonomic groups. In terms of mass spectra, high-resolution delivers greater space in the *m*/*z* domain, which would allow for more taxonomic resolution as long as the metabolite diversity correlates with the taxonomic diversity of the analyzed species. With a bigger single-cell profile database, the SPM algorithm would have to be simply optimized to avoid an increase in computation time.

## Conclusion

Here, we combined live-single cell mass spectrometry with an SPM approach in one workflow to reliably derive the taxonomic identity of a microalgal cell through its metabolic fingerprint. The analysis of single-cell spectra in both negative and positive polarity resulted in robust assignments of the taxonomic identity. Of the three tested similarity measures, *Eu* and *iEu* performed better in almost all test situations. The bootstrap-derived confidence scores improved the classification, mainly when applied to the more diverse mixed dataset (Fig. 5). The comparison of high-resolution spectra versus unit-mass resolution showed little gain in the success of the method at this early stage of the database development.

The herein described method demands no phycological expert knowledge, once a single-cell profiling database is established, ideally as an open-source repository. Since only cultures in well-defined conditions have been investigated, future studies will implement various stress conditions, as those were shown to have a strong influence on the metabolic profile of a microalgal cell (Faulkner et al. 2019; Driver et al. 2015). The present approach that monitors the metabolome has the potential to generate data about the physiological state of the single cells and thus about the metabolic heterogeneity of a plankton population. The discrimination of nutrient-depleted or aged cells could already be explained with single-cell metabolic profiling (Baumeister et al. 2019; Krismer et al. 2017). In future studies, a broader set of spectra that also takes into account the cells´ physiological condition could be implemented so that not only the cells identity but also its health status is revealed.

## Electronic supplementary material

Below is the link to the electronic supplementary material.

Supplementary file1 (DOCX 12561 kb)

## Data Availability

The spectra, R scripts and metadata reported in this paper are available via https://github.com/Pohnert-Lab/SC-MS-Identification
