# Supplementary material for: Identification to species level of live single microalgal cells from plankton samples with matrix-free laser/desorption ionization mass spectrometry
Source: Metabolomics. 2020 Feb 24;16(3):28. doi: 10.1007/s11306-020-1646-7 (PMC7036359; doi:10.1007/s11306-020-1646-7)
Supplement: Supplementary file 1 — Supplementary file1 (DOCX 12561 kb) [file 11306_2020_1646_MOESM1_ESM.docx]

**Supplementary information**

**for**

Identification to species level of live single microalgal cells from plankton samples with matrix-free laser/desorption ionization mass spectrometry

Tim U. H. Baumeister^1^, Marine Vallet^1^, Filip Kaftan^2^, Laure Guillou^3^, Aleš Svatoš^2*^, Georg Pohnert^1,4*^

^1^ Max Planck Institute for Chemical Ecology, Max Planck Fellow Group on Plankton Community Interaction, Hans-Knöll-Str. 8, 07745 Jena, Germany

^2^ Research Group Mass Spectrometry/Proteomics, Max Planck Institute for Chemical Ecology, Hans-Knöll-Str. 8, 07745 Jena, Germany

^3^ Sorbonne Université, CNRS, UMR7144 Adaptation et Diversité en Milieu Marin, Ecology of Marine Plankton (ECOMAP), Station Biologique de Roscoff SBR, 29680 Roscoff, France

^4^ Friedrich Schiller University Jena, Institute for Inorganic and Analytical Chemistry, Department of Bioorganic Analytics, Lessingstr. 8, 07743 Jena, Germany

* Corresponding Authors

georg.pohnert@uni-jena.de

svatos@ice.mpg.de

Table of content:

**Fig. S1** Representative pictures of the microalgal isolates from field samplings.

**Table S1** Summary of the identity and single-cell profiles acquired from the microalgal samples.

**Fig. S2** Boxplots of the number of peaks (logarithmic scale), grouped by **genus** (**mixed dataset**) and quality of underlying spectra. Median values (absolute numbers) and the total number of spectra is shown.

**Fig. S3** Boxplots of the number of peaks per spectrum (logarithmic scale), grouped by **species** (**collection strain dataset**), quality of underlying spectra, and polarity. Median values (absolute numbers) and the total number of spectra is shown.

**Fig. S4** Frequencies of **positive** *m*/*z* values (bin size *m*/*z* 10) per spectrum, grouped by **genus** (**mixed dataset**).

**Fig. S5** Frequencies of **negative** *m*/*z* values (bin size *m*/*z* 10) per spectrum, grouped by **genus** (**mixed dataset**).

**Fig. S6** Frequencies of **positive** *m*/*z* values (bin size *m*/*z* 10) per spectrum, grouped by **species** (**collection strain dataset**).

**Fig. S7** Frequencies of **negative** *m*/*z* values (bin size *m*/*z* 10) per spectrum, grouped by **species** (**collection strain dataset**).

**Table S2** Sensitivity and error rates at a threshold score at the genus and species level further divided by both polarities, taxonomy level and dataset. Conf(score) indicates a confidence score based on the used similarity score. Values obtained for confidence score are italicized.

**Fig. S8** Confusion matrices of identification results of microalgae at the **genus level** by *Cos*, *Eu*, and *iEu*, based on the **mixed dataset**. (**a**) Underlying high-resolution spectra were obtained in positive polarity. (**b**) Underlying **high-resolution spectra** were obtained in negative polarity. Confidence intervals (CI 95%) overall accuracy are indicated above each plot.

**Fig. S9** Confusion matrices of identification results of microalgae at the **genus level** by *Cos*, *Eu*, and *iEu* using **integer mass spectra** derived from the **mixed dataset** in positive (**a**) and negative (**b**) polarities.

**Fig. S10** Confusion matrices of identification results of microalgae at the **species level** by *Cos*, *Eu*, and *iEu* using **integer mass spectra** derived from the **collection strain dataset** in positive (**a**) and negative (**b**) polarities.

**Fig. S11** Receiver operating characteristic curves and corresponding area under curves (AUC) values of taxonomic identifications of microalgae at the **genus level** using **integer mass spectra** for the **mixed dataset** by use of Cos, Eu, and iEu (**a**, **c**) and combined with bootstrap assessment (**b**, **d**).

**Fig. S12** Receiver operating characteristic curves and corresponding area under curves (AUC) values of taxonomic identifications of microalgae at the **species level** using **integer mass spectra** for the **collection strain dataset** by use of Cos, Eu, and iEu (**a**, **c**) and combined with bootstrap assessment (**b**, **d**).

**Datafile S1** Identification results at the **genus level** of **high-resolution mass spectra** from the **collection strain dataset** acquired in **positive polarity** (separate CSV file on GitHub).

**Datafile S2** Identification results at the **genus level** of **high-resolution mass spectra** from the **collection strain dataset** acquired in **negative polarity spectra** (separate CSV file on GitHub).

**Data file S3** Identification results at the **species level** of **high-resolution mass spectra** from the **collection strain dataset** acquired in **positive polarity spectra** (separate CSV file on GitHub).

**Data file S4** Identification results at the **species level** of **high-resolution mass spectra** from the **collection strain dataset** acquired in **negative polarity spectra** (separate CSV file on GitHub).

**Data file S5** Identification results at the **genus level** of **high-resolution mass spectra** from the **mixed dataset** acquired in **positive polarity** (separate CSV file on GitHub).

**Data file S6** Identification results at the **genus level** of **high-resolution mass spectra** from the **mixed dataset** acquired in **negative polarity spectra** (separate CSV file on GitHub).

**Data file S7** Identification results at the **genus level** of **integer mass spectra** derived from the **mixed dataset** acquired in **positive polarity** (separate CSV file on GitHub).

**Data file S8** Identification results at the **genus level** of **integer mass spectra** acquired in **negative polarity** derived from the **mixed dataset** (separate CSV file on GitHub).

**Data file S9** Identification results at the **species level** of **integer mass spectra** acquired in **positive polarity** derived from the **collection strain dataset** (separate CSV file on GitHub).

**Data file S10** Identification results at the **species level** of **integer mass spectra** acquired in **negative polarity** derived from the **collection strain dataset** (separate CSV file on GitHub)

**Data file S11** Metadata of single-cell spectra (**mixed dataset**, **genus level**, **positive polarity**). Separate CSV file on GitHub.

**Data file S12** Metadata of single-cell spectra (**mixed dataset**, **genus level**, **negative polarity**). Separate CSV file on GitHub.

**Data file S13** Metadata of single-cell spectra (**collection strain dataset**, **species level**, **positive polarity**). Separate CSV file on GitHub.

**Data file S14** Metadata of single-cell spectra (**collection strain dataset**, **species level**, **negative polarity**). Separate CSV file on GitHub.

**R scripts** that were used to obtain datasets S1-S10 (identification results) are available on GitHub (<https://github.com/Pohnert-Lab/SC-MS-Identification>). Instructions how to reproduce the herein shown results (confusion matrices, ROC curves, and supplementary figures S2-S7) are available on GitHub. Briefly explained, only the R scripts to the corresponding datafiles S1-S10 need to be downloaded from GitHub and have to be executed in R. Per default, the scripts will obtain the spectra (as .RData) from GitHub automatically and rerun the whole analysis, starting with the spectral pattern matching (SPM). Optionally, SPM can be skipped, and the plots will be generated from the datafiles S1-S10. Therefore, the line that imports the dataset from GitHub has to be uncommented, i.e. activated and the preceding SPM lines can be ignored.

**Single-cell spectra**: Blank subtracted and de-noised spectra are available as individual CSV files and combined as RData-files on GitHub.


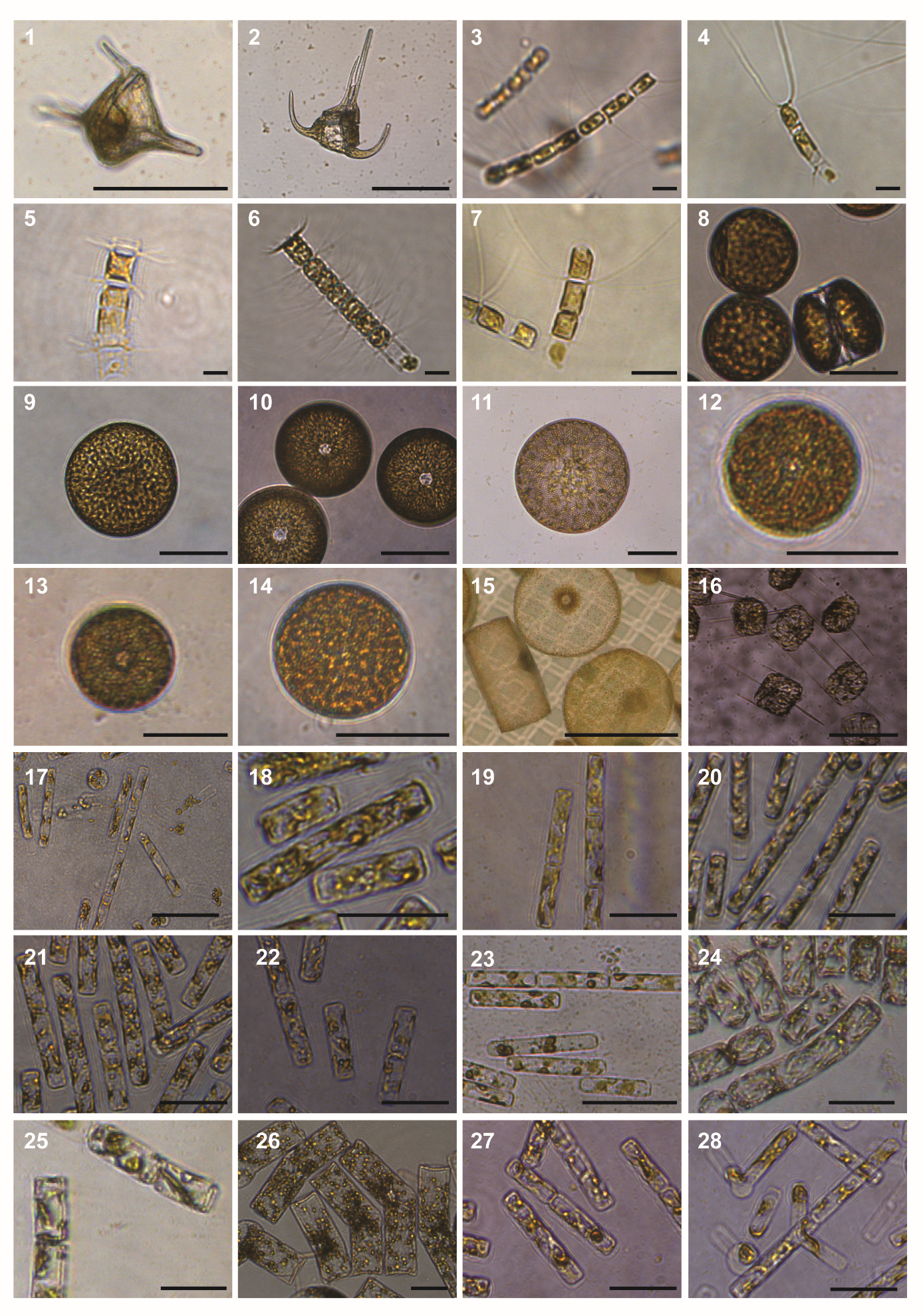


**
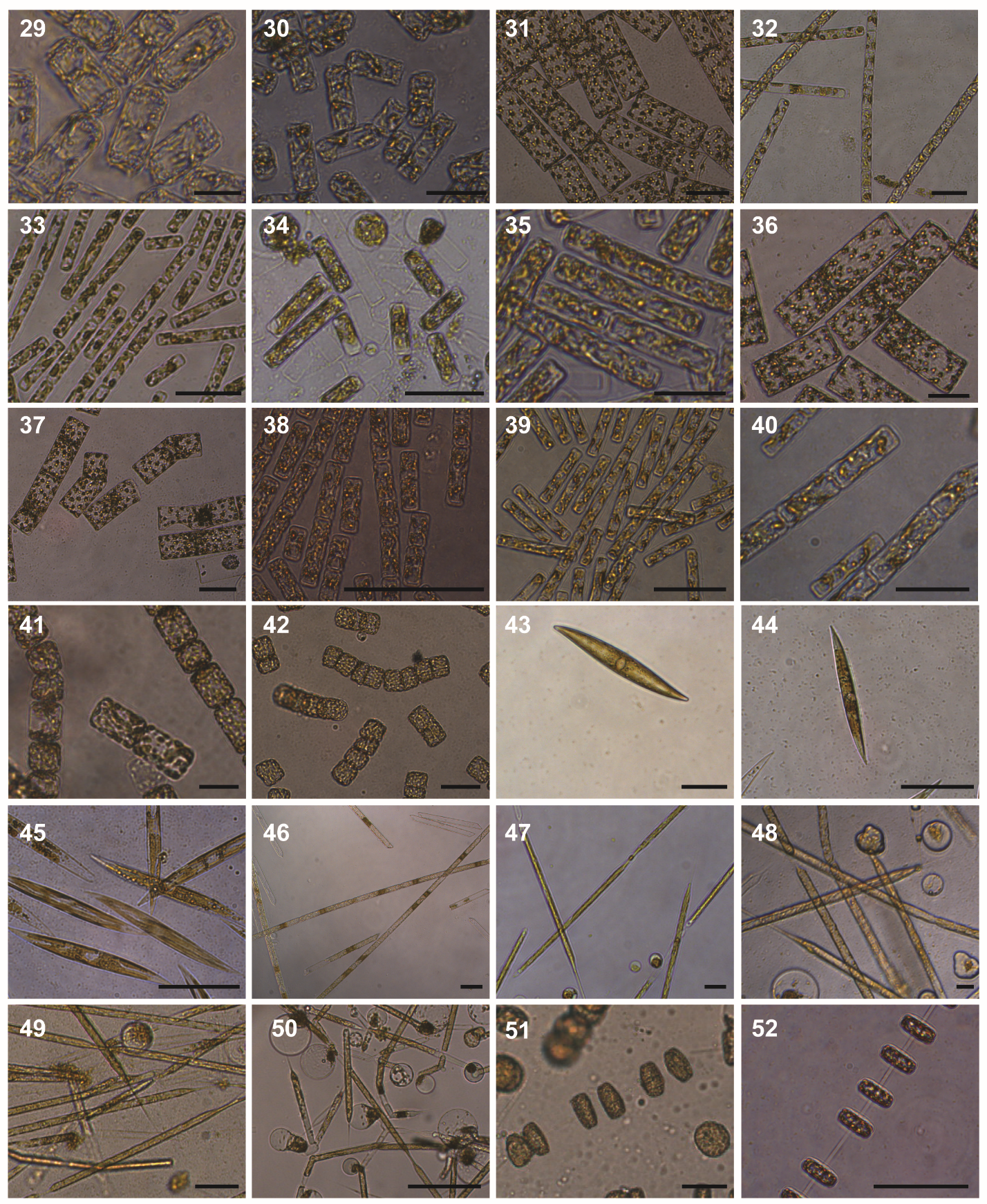
**

**Fig. S1** Representative pictures of the microalgal isolates from field samplings. *Ceratium* sp. 1) G-P9-6, 2) Norway2017. Scale bars equal to 50 µm. *Chaetoceros* sp. 3) G-P9-6, 4) G-P4-4, 5) G-P6-3, 6) G-P7-4, 7) G-P9-1. Scale bars equal to 10 µm. *Coscinodiscus granii* 8) Helg2016, 9) Helg2017. *Coscinodiscus radiatus* 10) Helg2017. Scale bars equal to 20 µm. *Coscinodiscus* sp. 11) Norway2017, 12) R-R655, 13) R-R2512, 14) R-R4326. Scale bars equal to 20 µm. *Coscinodiscus wailesii* 15) Helg2016. Scale bar equals to 250 µm. *Ditylum* sp. 16) R-R1. Scale bar equals to 50 µm. *Guinardia* sp. 17) R-R252, 18) R-R1417, 19) R-R1419, 20) R-R1518, 21) R-R1524, 22) R-R2523, 23) R-R2525, 24) R-R142, 25) R-R143, 26) R-R149, 27) R-R155, 28) R-R158, 29) R-R256, 30) R-R258, 31) R-R442, 32) R-R452, 33) R-R557, 34) R-R1412, 35) R-R1414, 36) R-R1427, 37) R-R1428, 38) R-R1509, 39) R-R1511, 40) R-R2522. Scale bars equal to 20 µm. *Lauderia* sp. 41) R-R4511, 42) R-R4513. Scale bars equal to 20 µm. *Pleurosigma* sp. 43) R-PS1, 44) R-R653, 45) R-2510. Scale bars equal to 25 µm. *Rhizosolenia* sp. 46) R-R141, 47) R-R154, 48) R-R257, 49) R-R2511, 50) R-R4518. Scale bars equal to 40 µm. *Thalassiosira* sp. 51) R-R451, 52) R-R459. Scale bars equal to 25 µm.

**Table S1** Summary of the identity and single-cell profiles acquired from the microalgal samples. The taxonomic identity, sampling site and type of strain (origin from culture collection or field sampling) are presented along with the number of single-cell profiles acquired per algal isolate or strain in positive (+) and negative (-) polarities.

| Genus | Species | Nr. of spectra  (+) | Nr. of spectra  (−) | Isolation site | Strain type | Cell size (µm) | Year of analysis | Nr. of strains |
| --- | --- | --- | --- | --- | --- | --- | --- | --- |
| *Ceratium* | sp. | NA | 2 | Farsund, Norway | Field sampling | 100 | 2018 | 1 |
| *Ceratium* | sp. | 41 | 24 | Leros, Greece | Field sampling | 50 | 2018,  2019 | 1 |
| *Chaetoceros* | cf. *wighamii* | 9 | 4 | Denmark | Collection | 10 | 2018 | 1 |
| *Chaetoceros* | *diadema* | 16 | 16 | Roscoff, France | Collection | 10 | 2018 | 1 |
| *Chaetoceros* | *didymus* | 17 | 9 | Italy | Field sampling | 10 | 2018 | 1 |
| *Chaetoceros* | sp. | 59 | 21 | Leros, Greece | Field sampling | 10 | 2018 | 5 |
| *Chaetoceros* | sp. | 17 | 17 | Penzé, France | Field sampling | 10 | 2019 | 1 |
| *Coscinodiscus* | *granii* | 17 | NA | Denmark | Collection | 50 | 2018 | 1 |
| *Coscinodiscus* | *granii* | 23 | 25 | Helgoland, Germany | Field sampling | 30 | 2018,  2019 | 2 |
| *Coscinodiscus* | *radiatus* | 18 | 16 | Helgoland, Germany | Field sampling | 50 | 2018,  2019 | 2 |
| *Coscinodiscus* | sp. | 15 | 16 | Farsund, Norway | Field sampling | 50 | 2018 | 1 |
| *Coscinodiscus* | sp. | 3 | NA | Penzé, France | Field sampling | 20 | 2018 | 2 |
| *Coscinodiscus* | *wailesii* | 14 | 17 | Helgoland, Germany | Field sampling | 250 | 2018 | 1 |
| *Ditylum* | sp. | 19 | 33 | Penzé, France | Field sampling | 86 | 2018,  2019 | 1 |
| *Guinardia* | *flaccida* | 6 | NA | Roscoff, France | Collection | NA | 2018 | 1 |
| *Guinardia* | sp. | 26 | 24 | Penzé, France | Field sampling | 10-30 | 2018 | 26 |
| *Lauderia* | *annulata* | 6 | NA | Roscoff, France | Collection | 30 | 2018 | 1 |
| *Lauderia* | sp. | 6 | 5 | Penzé, France | Field sampling | 20 | 2018 | 2 |
| *Pleurosigma* | sp. | 25 | 23 | Penzé, France | Field sampling | 90 | 2018,  2019 | 3 |
| *Rhizosolenia* | sp. | 22 | 19 | Penzé, France | Field sampling | 120 | 2018,  2019 | 5 |
| *Thalassiosira* | *punctigera* | 11 | NA | Roscoff, France | Collection | 35 | 2018 | 1 |
| *Thalassiosira* | sp. | 4 | 8 | Penzé, France | Field sampling | 20-30 | 2018 | 3 |
| *Thalassiosira* | sp. | 9 | NA | Roscoff, France | Collection | 20 | 2018 | 1 |

NA: data not available.


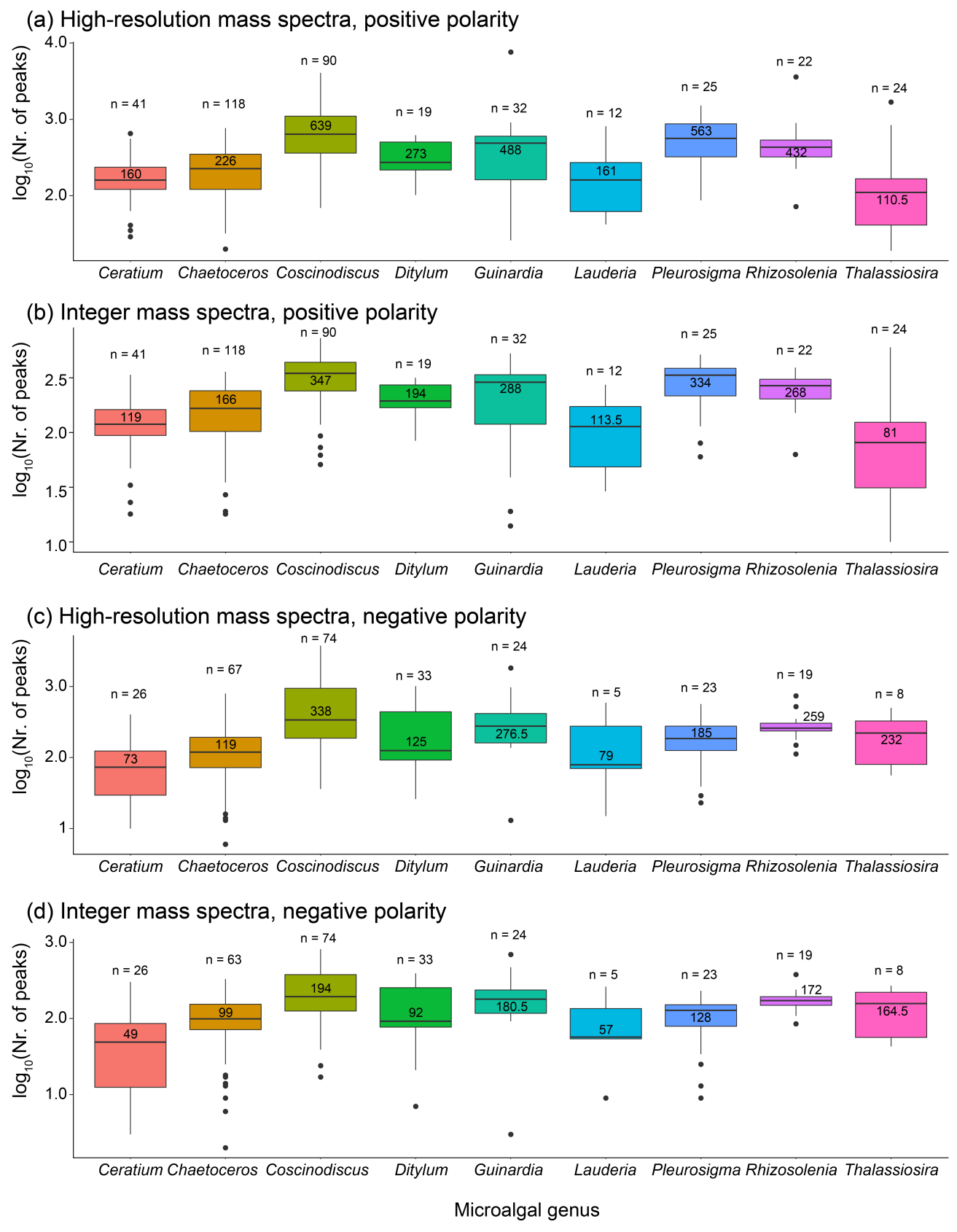


**Fig. S2** Boxplots of the number of peaks (logarithmic scale), grouped by **genus** (**mixed dataset**) and quality of underlying spectra. Median values (absolute numbers) and the total number of spectra is shown.


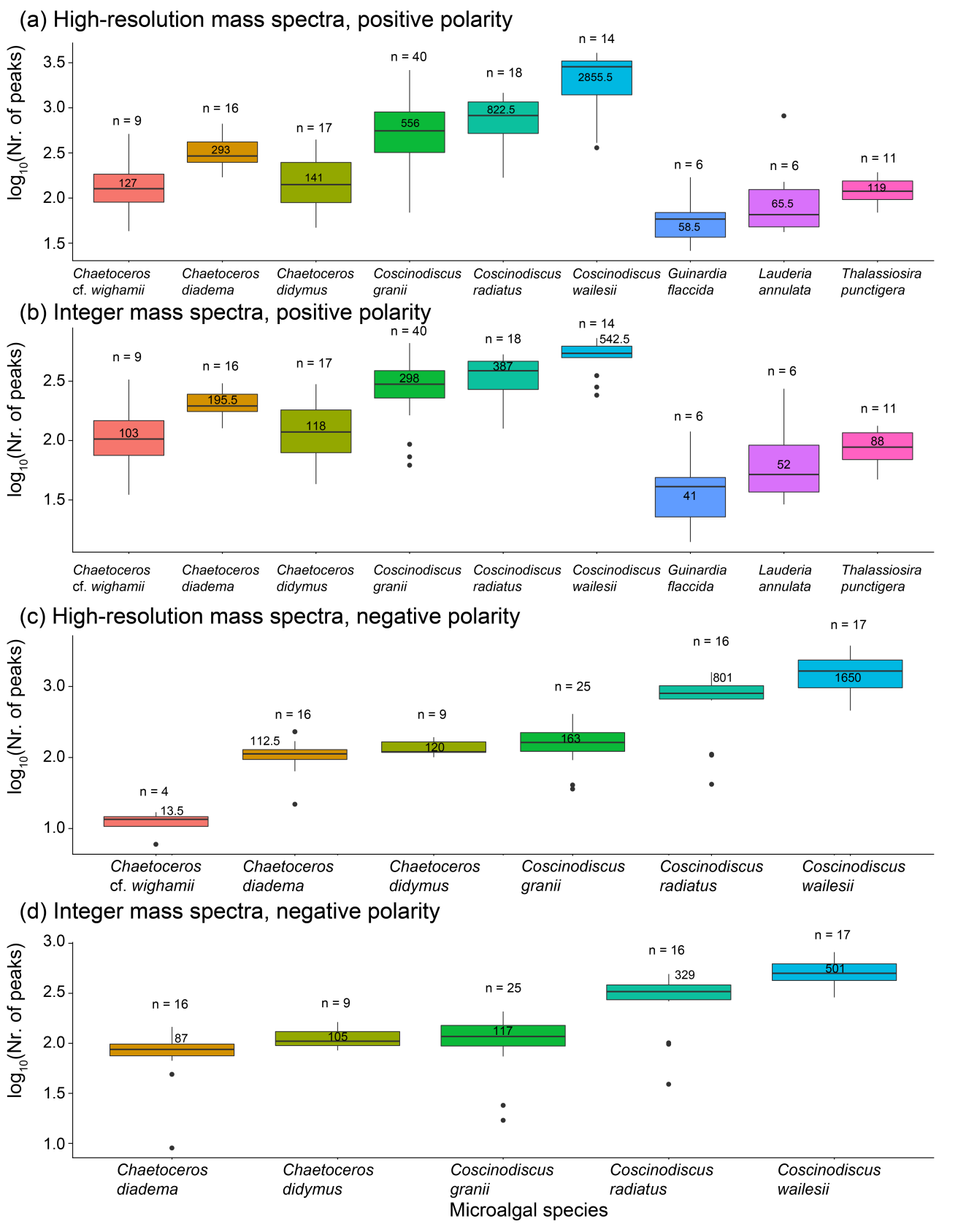


**Fig. S3** Boxplots of the number of peaks per spectrum (logarithmic scale), grouped by **species** (**collection strain dataset**), quality of underlying spectra, and polarity. Median values (absolute numbers) and the total number of spectra is shown.


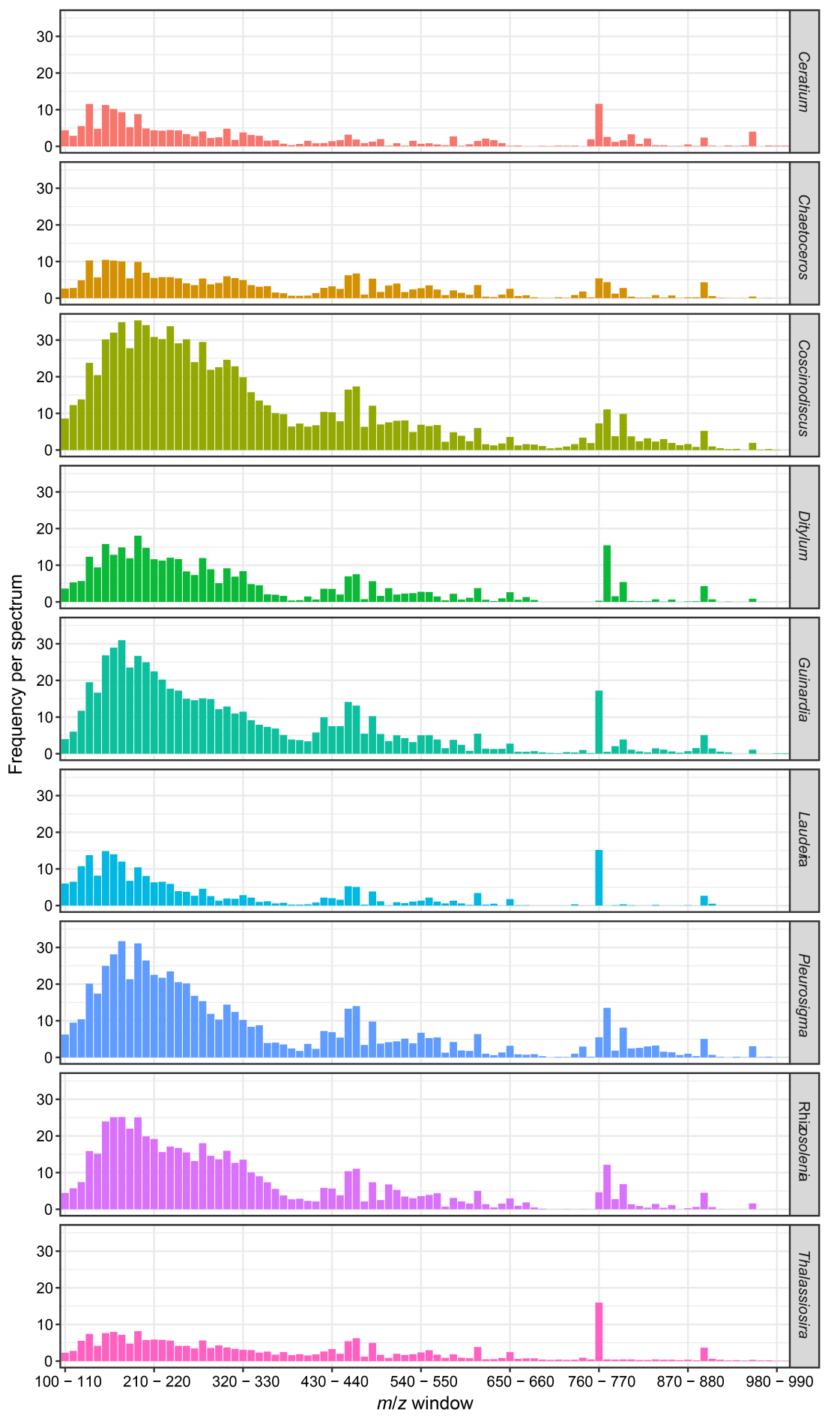


**Fig. S4** Frequencies of **positive** *m*/*z* values (bin size *m*/*z* 10) per spectrum, grouped by **genus** (**mixed dataset**).


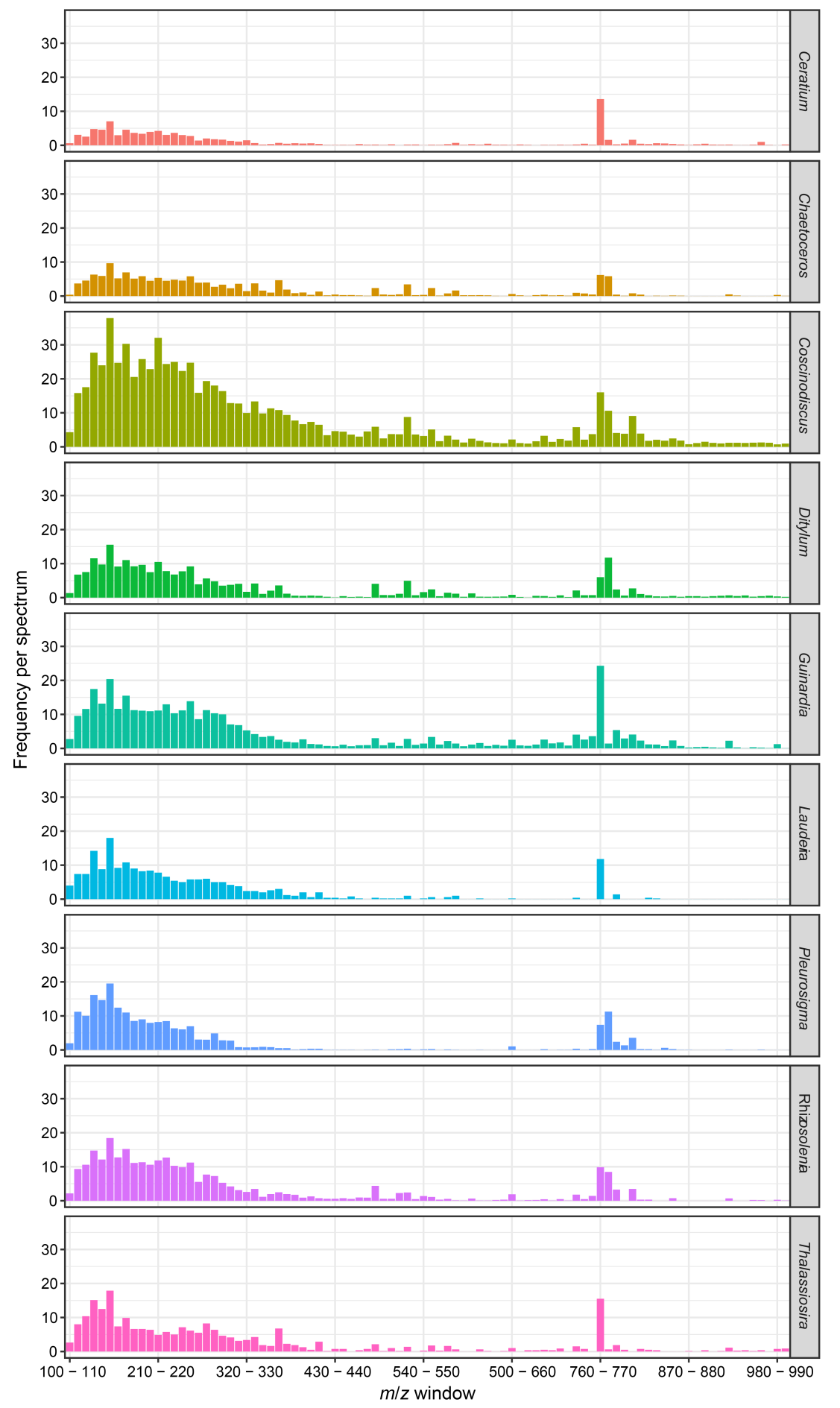


**Fig. S5** Frequencies of **negative** *m*/*z* values (bin size *m*/*z* 10) per spectrum, grouped by **genus** (**mixed dataset**).


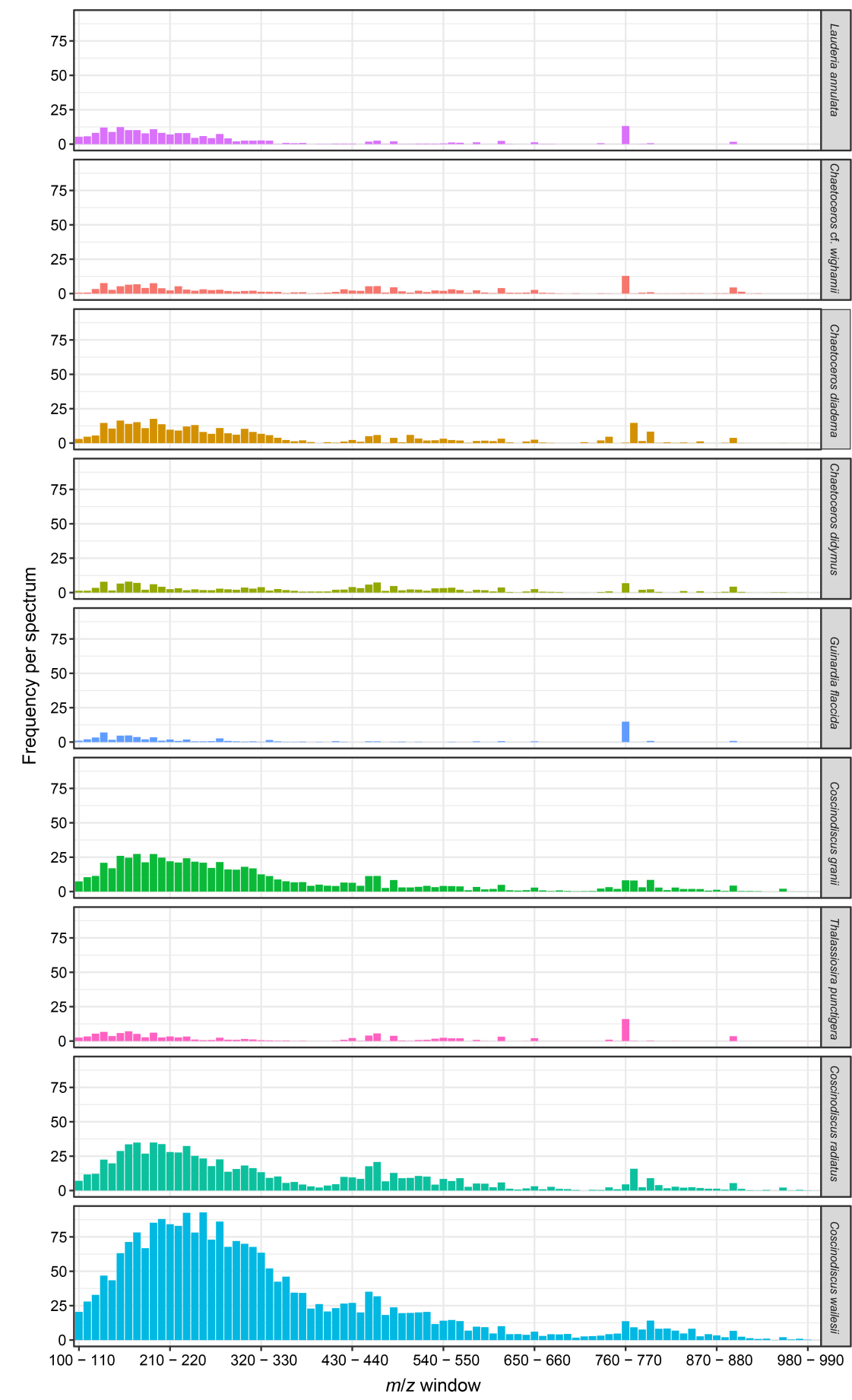


**Fig. S6** Frequencies of **positive** *m*/*z* values (bin size *m*/*z* 10) per spectrum, grouped by **species** (**collection strain dataset**).


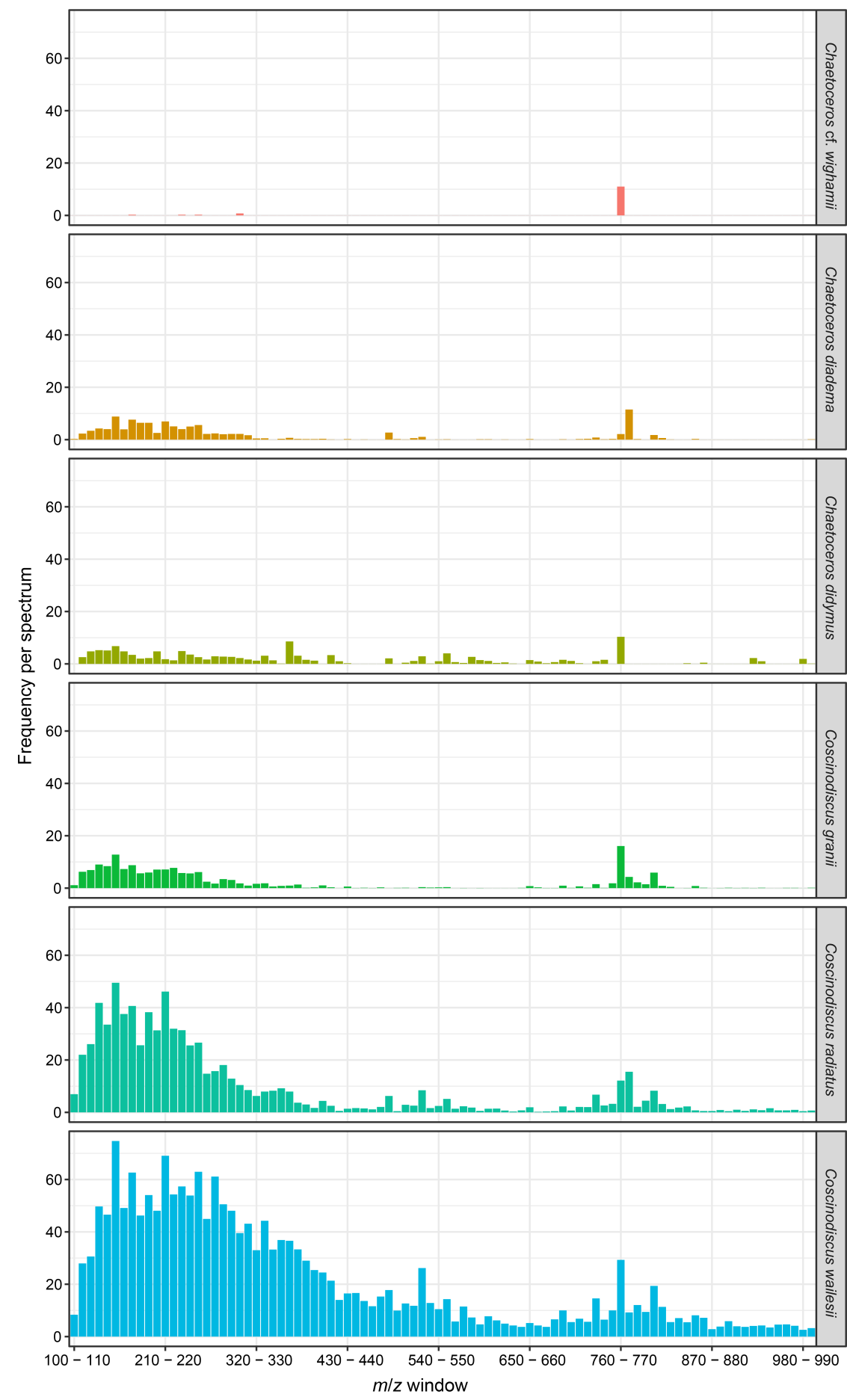


**Fig. S7** Frequencies of **negative** *m*/*z* values (bin size *m*/*z* 10) per spectrum, grouped by **species** (**collection strain dataset**).

**Table S2** Sensitivity and error rates at a threshold score at the genus and species level further divided by both polarities, taxonomy level and dataset. Conf(score) indicates a confidence score based on the used similarity score. Values obtained for confidence score are italicized.

| Dataset | Level | Polarity | Scoring measure | Threshold score | Error rate | Sensitivity |
| --- | --- | --- | --- | --- | --- | --- |
| Collection strain dataset | Genus | Positive | *Cos* | 0.35 | 0.04 | 1.00 |
|  |  |  | *Eu* | 0.00 | 0.04 | 1.00 |
|  |  |  | *iEu* | 0.00 | 0.04 | 1.00 |
|  |  |  | Conf(*Cos*) | *0.44* | *0.04* | *1.00* |
|  |  |  | Conf(*Eu*) | *0.00* | *0.04* | *1.00* |
|  |  |  | Conf(*iEu*) | *0.00* | *0.04* | *1.00* |
|  |  | Negative | *Cos* | 0.00 | 0.02 | 1.00 |
|  |  |  | *Eu* | 0.00 | 0.01 | 1.00 |
|  |  |  | *iEu* | 0.00 | 0.05 | 1.00 |
|  |  |  | Conf(*Cos*) | *0.00* | *0.02* | *1.00* |
|  |  |  | Conf(*Eu*) | *0.00* | *0.01* | *1.00* |
|  |  |  | Conf(*iEu*) | *0.00* | *0.05* | *1.00* |
| Collection strain dataset | Species | Positive | *Cos* | 0.98 | 0.00 | 0.05 |
|  |  |  | *Eu* | 0.25 | 0.05 | 0.68 |
|  |  |  | *iEu* | 0.62 | 0.05 | 0.62 |
|  |  |  | Conf(*Cos*) | *0.81* | *0.04* | *0.39* |
|  |  |  | Conf(*Eu*) | *1.00* | *0.05* | *0.69* |
|  |  |  | Conf(*iEu*) | *0.98* | *0.04* | *0.79* |
|  |  | Negative | *Cos* | 0.70 | 0.04 | 0.91 |
|  |  |  | *Eu* | 0.00 | 0.05 | 1.00 |
|  |  |  | *iEu* | 0.42 | 0.03 | 0.90 |
|  |  |  | Conf(*Cos*) | *0.51* | *0.05* | *0.95* |
|  |  |  | Conf(*Eu*) | *0.00* | *0.05* | *1.00* |
|  |  |  | Conf(*iEu*) | *0.69* | *0.05* | *0.99* |
| Mixed dataset | Genus | Positive | *Cos* | 0.88 | 0.10 | 0.80 |
|  |  |  | *Eu* | 0.26 | 0.10 | 0.71 |
|  |  |  | *iEu* | 0.60 | 0.09 | 0.63 |
|  |  |  | Conf(*Cos*) | *0.39* | *0.10* | *0.95* |
|  |  |  | Conf(*Eu*) | *0.85* | *0.10* | *0.89* |
|  |  |  | Conf(*iEu*) | *0.60* | *0.10* | *0.98* |
|  |  | Negative | *Cos* | 0.79 | 0.10 | 0.85 |
|  |  |  | *Eu* | 0.18 | 0.10 | 0.87 |
|  |  |  | *iEu* | 0.48 | 0.10 | 0.84 |
|  |  |  | Conf(*Cos*) | *0.48* | *0.10* | *0.86* |
|  |  |  | Conf(*Eu*) | *0.77* | *0.10* | *0.90* |
|  |  |  | Conf(*iEu*) | *0.65* | *0.10* | *0.96* |

**
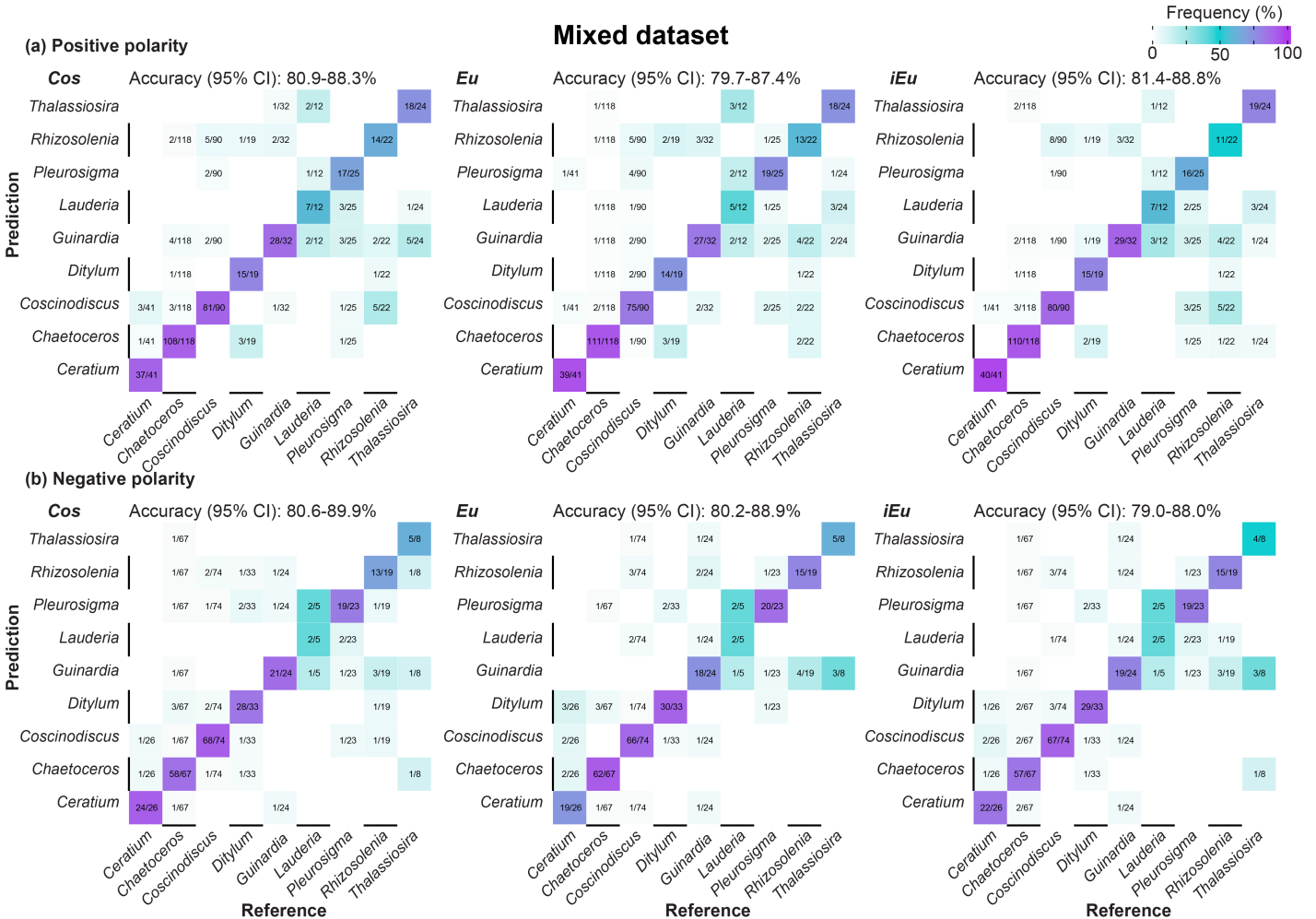
**

**Fig. S8** Confusion matrices of identification results of microalgae at the **genus level** by *Cos*, *Eu*, and *iEu* (Yang *et al*. 2017), for the **mixed dataset**. (**a**) Underlying high-resolution spectra were obtained in positive polarity. (**b**) Underlying **high-resolution spectra** were obtained in negative polarity. Confidence intervals (CI 95%) of overall accuracy are indicated above each plot.


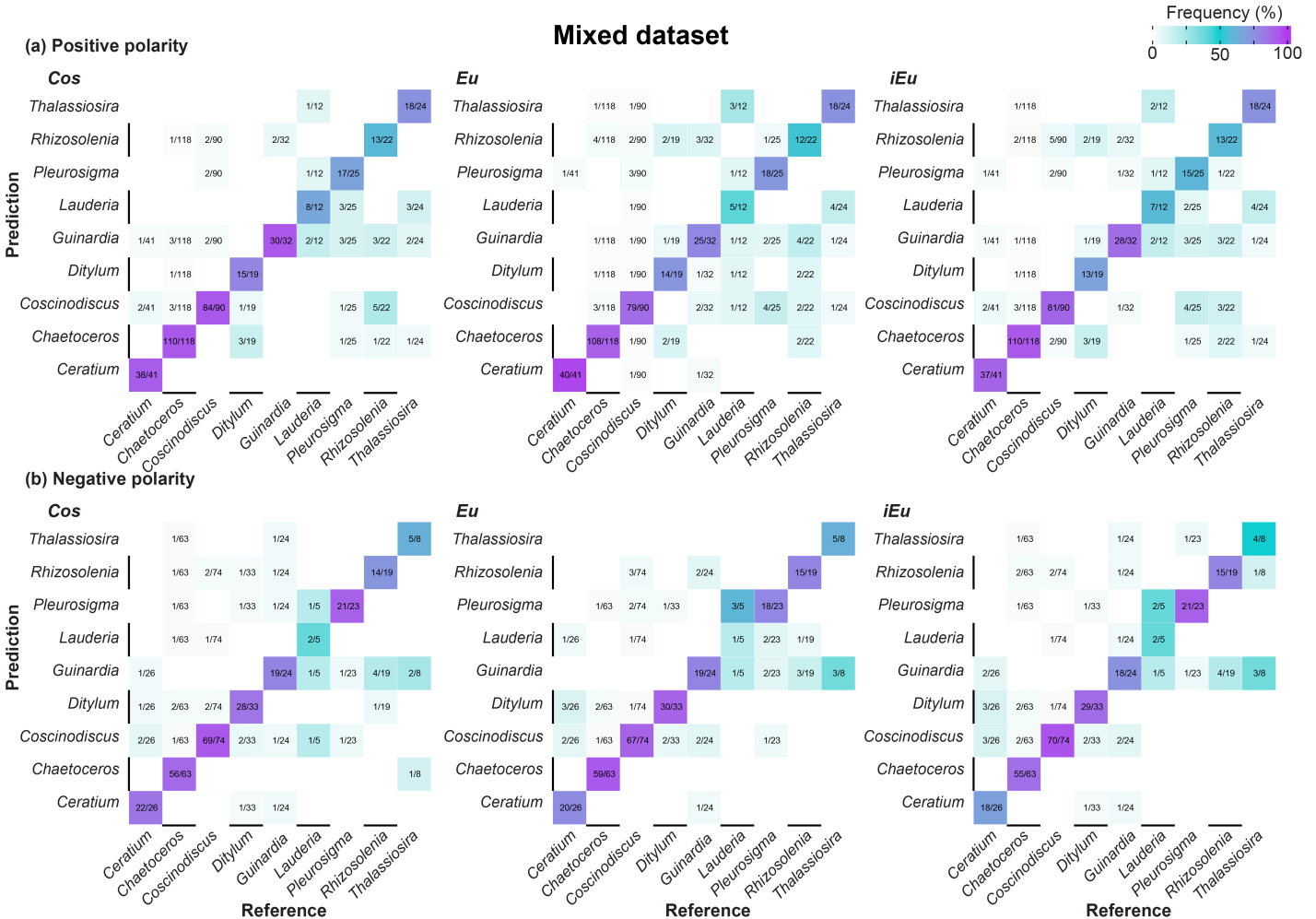


**Fig. S9** Confusion matrices of identification results of microalgae at the **genus level** by *Cos*, *Eu*, and *iEu* (Yang *et al*. 2017), using **integer mass spectra** for the **mixed dataset** in positive (**a**) and negative (**b**) polarity.


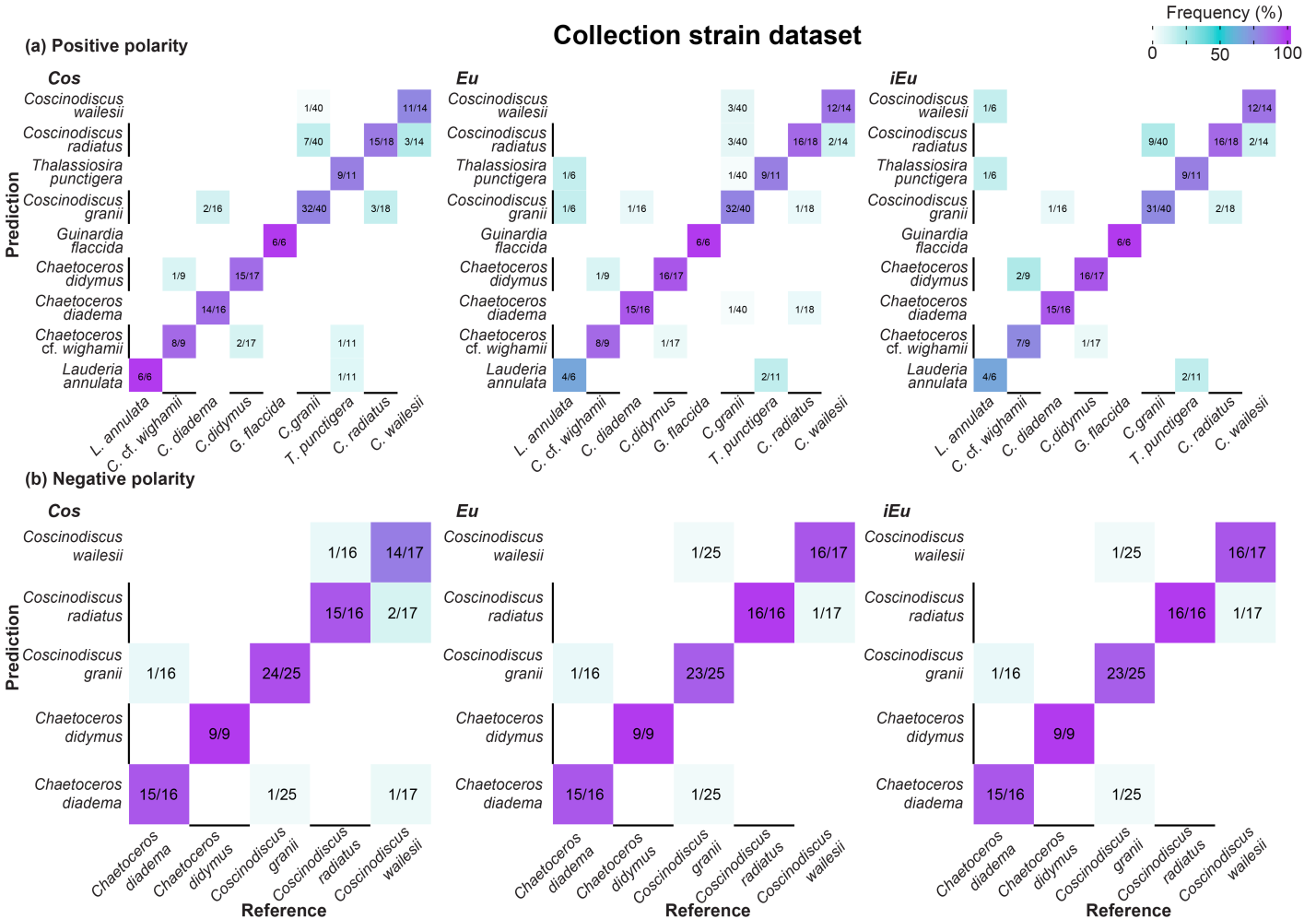


**Fig. S10** Confusion matrices of identification results of microalgae at the **species level** by *Cos*, *Eu* , and *iEu* (Yang *et al*. 2017), using **integer mass spectra** for the **collection strain dataset** in positive (**a**) and negative (**b**) polarity.


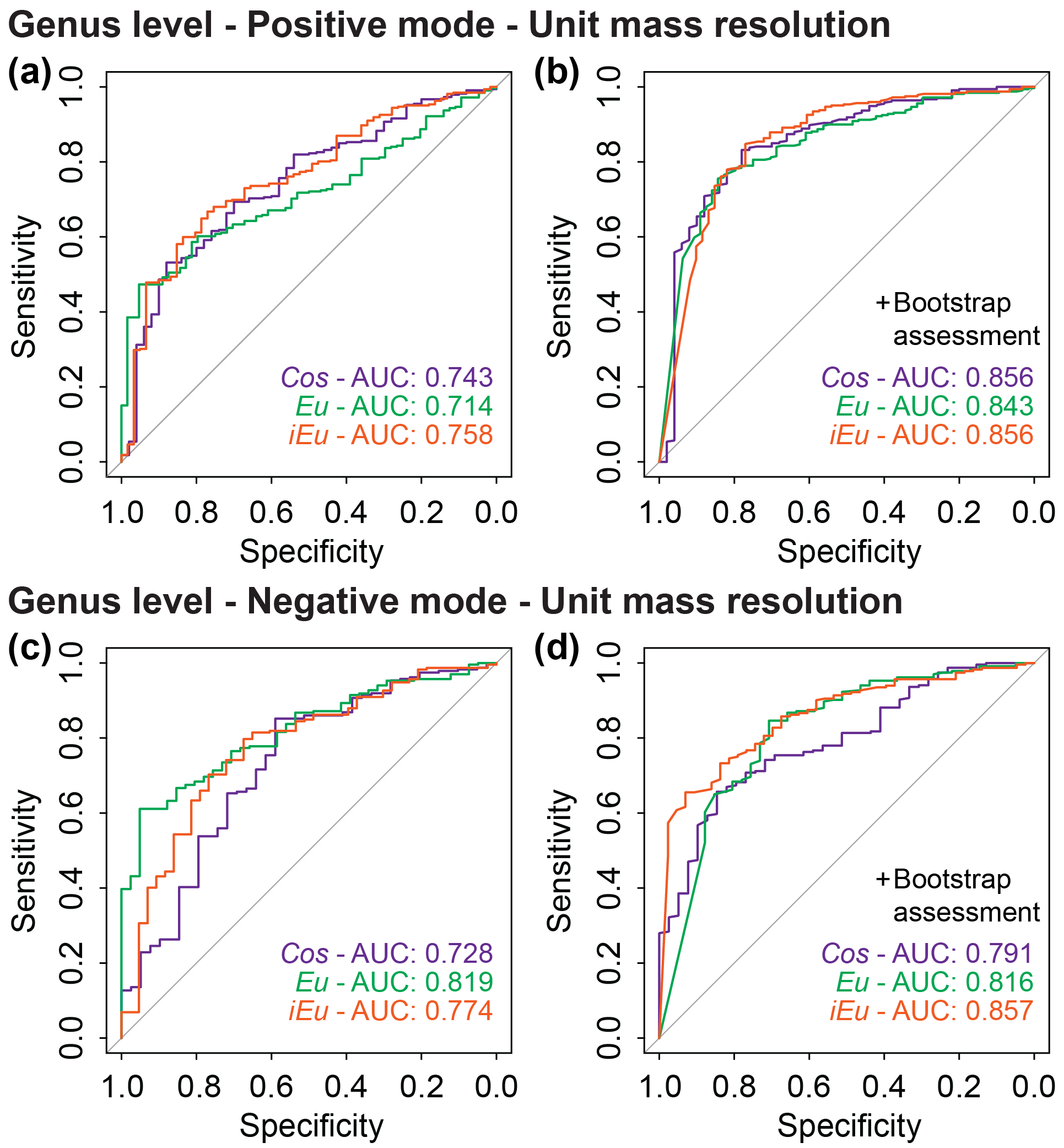


**Fig. S11** Receiver operating characteristic curves and corresponding area under curves (AUC) values of taxonomic identifications of microalgae at the **genus level** using **integer mass spectra** for the **mixed dataset** by use of *Cos*, *Eu*, and *iEu* (Yang *et al*. 2017) (**a**, **c**) and combined with bootstrap assessment (**b**, **d**). The AUC curves obtained for each classifier (*Cos*, *Eu*, *iEu*) analyzed are indicated in purple, green or orange color, respectively.


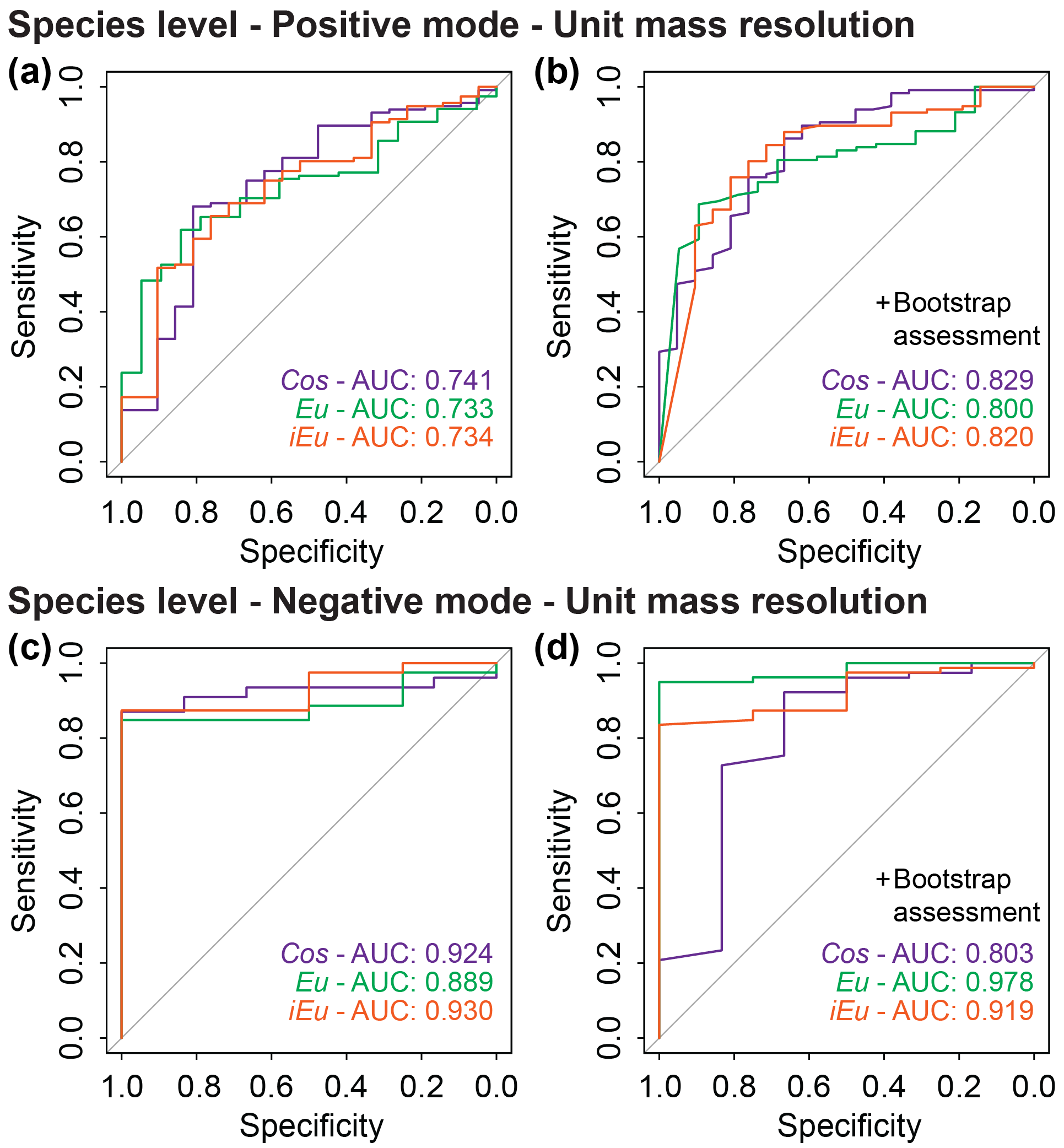


**Fig. S12** Receiver operating characteristic curves and corresponding area under curves (AUC) values of taxonomic identifications of microalgae at the **species level** using **integer mass spectra** for the **collection strain dataset** by use of *Cos*, *Eu*, and *iEu* (Yang *et al*. 2017) (**a**, **c**) and combined with bootstrap assessment (**b**, **d**). The AUC curves obtained for each classifier (*Cos*, *Eu*, *iEu*) analyzed are indicated in purple, green or orange color, respectively.
